# Determinants of Low Bone Turnover in Type 2 Diabetes-the Role of PTH

**DOI:** 10.1007/s00223-022-01022-7

**Published:** 2022-10-03

**Authors:** Janina Vavanikunnel, Lilian Sewing, Maria Triantafyllidou, Anna Steighardt, Sandra Baumann, Andrea Egger, Leticia Grize, Barbara Felix, Marius Kraenzlin, Christoph Henzen, Christian Meier

**Affiliations:** 1 Division of Endocrinology, Diabetes and Metabolism, University Hospital Basel, Basel, Switzerland; 2 Department of Internal Medicine, Kantonsspital Lucerne, Switzerland; 3 Swiss Tropical and Public Health Institute, Basel, Switzerland; 4 University of Basel, Basel, Switzerland; 5 Division of Endocrinology, Kantonsspital Baselland, Switzerland; 6 Endocrine Clinic and Laboratory, Basel, Switzerland

**Keywords:** Diabetes, Hypoparathyroidism, Bone turnover

## Abstract

Determinants of low bone turnover in type 2 diabetes (T2DM) are poorly understood. To investigate the relationship between markers of bone turnover, glycaemic control, disease duration and calciotropic hormones in T2DM we assessed baseline biochemical data from the DiabOS Study, a prospective multicenter observational cohort study. In a cross-sectional study-design data from 110 postmenopausal women and men aged 50–75 years diagnosed with T2DM for at least 3 years and 92 non-diabetic controls were evaluated. Biochemical markers of bone formation (N-terminal propeptide of type I procollagen [PINP]), bone-specific alkaline phosphatase [BAP]) and resorption (C-terminal cross-linking telopeptide of type I collagen [CTX]), measures of calcium homeostasis (intact parathormone [iPTH], 25-Hydroxyvitamin D, calcium, magnesium) and glycaemic control were assessed. After adjustment for age, gender and body mass index (BMI), patients with T2DM had lower serum levels of PINP (*p* < 0.001), CTX (*p* < 0.001), iPTH (*p* = 0.03) and magnesium (*p* < 0.001) compared to controls. Serum calcium, creatinine, 25-Hydroxyvitamin D and sclerostin did not differ between both groups. In multivariate linear regression analyses only serum iPTH remained an independent determinant of bone turnover markers in T2DM (PINP: *p* = 0.02; CTX: *p* < 0.001 and BAP: *p* < 0.01), whereas glycated haemoglobin (HbA1c), disease duration, age and BMI were not associated with bone turnover. In conclusion low bone turnover in T2DM is associated with low iPTH. The underlying mechanism remains to be elucidated.

## Introduction

Type 2 diabetes mellitus (T2DM) has been established to be a state of low bone turnover [1–6]. Histological data indicate significant reductions in indices of bone resorption [2] as well as reduced dynamic indices of bone formation [2, 3]. This has been confirmed in various clinical studies assessing biochemical markers of bone turnover [2, 3, 7–10]. Based on a meta-analysis Hygum et al. found that both markers of bone formation (N-terminal propeptide of type I procollagen (PINP) and osteocalcin (OC)) and markers of bone resorption (C-terminal cross-linking telopeptide of type I collagen (CTX) and isoform 5b of tartrate resistant acid phosphatase (TRACP5b)) were decreased in T2DM [11].

Type 2 diabetes is associated with an increased risk for fragility fractures [12–15]; however, the mechanisms accounting for bone fragility remain to be elucidated. Since patients with T2DM have equivalent or higher bone mineral density (BMD) as compared to non-diabetic controls (CO) [16, 17], the differences in BMD do not explain the increased fracture risk in T2DM [11]. Therefore, other determinants need to be identified [8]. It has been proposed that a compromised bone quality is ultimately responsible for fragility fractures in T2DM and altered bone remodelling is being discussed in this context [18]. Whether the increased fracture risk might be connected to an accumulation of microfractures facilitated by low bone turnover [19] is yet to be demonstrated. The role of low bone turnover for prediction of fracture risk in T2DM is unclear: A recent prospective case–control study reported that low bone turnover does not predict incident fracture risk in older adults with T2DM [20]. On the other hand, an earlier study in diabetic Japanese postmenopausal women showed that those patients with lower bone formation markers had a significantly higher vertebral fracture risk [21].

Overall, the pathophysiological mechanisms underlying low bone turnover in T2DM are poorly understood. A state of relative hypoparathyroidism has been suggested to contribute to low bone turnover in patients with diabetes: Reduced serum levels of CTX and TRAP5b have been found to correlate with low levels of parathormone (PTH) but potential implications of serum magnesium levels were not addressed [21, 22]. An impaired PTH secretion caused by a calcium-sensing defect or secondary to chronic hypomagnesemia has been discussed in T2DM [23] and an increased prevalence of hypomagnesemia has been identified in T2DM, especially in patients with poor glycaemic control, with a longer disease duration and chronic vascular complications [24, 25].

However, the chronic hyperglycaemic state itself may decrease bone turnover [26]. Elevated plasma glucose was associated with decreased levels of CTX, P1NP and OC [27] in patients with diabetes and negative correlations between levels of glycated haemoglobin (HbA1c) and OC and CTX have been observed [16, 28]. Furthermore, serum levels of sclerostin, a negative regulator of osteoblastogenesis, were elevated in T2DM [10, 29] and have been shown to correlate with the occurrence of fragility fractures [30, 31].

Further insight into the determinants of low bone turnover may improve the detection and treatment of patients with diabetic bone disease: To our knowledge this is the first study to comprehensively evaluate potential regulators of low bone turnover in type 2 diabetes including iPTH, magnesium, glycaemic control, disease duration and sclerostin levels.

## Methods

### Study Population and Design

Data were obtained from the DiabOS Study, a prospective multicenter observational cohort study evaluating skeletal health in T2DM and non-diabetic controls. The study was conducted according to the ethical guidelines of the Declaration of Helsinki, the International Conference on Harmonization guidelines on good clinical practice and national legal and regulatory requirements and was approved by the local ethical committee. Written informed consent was obtained from all patients and non-diabetic controls.

Postmenopausal women and men (aged 50–75 years, body mass index [BMI] 18–37 kg/m^2^) and postmenopausal female and male non-diabetic controls were recruited at University Hospital Basel, Kantonsspital Luzern and Kantonsspital Baselland, Switzerland and via press advertisement. Patients with diabetes were enrolled if they had a documented diagnosis of type 2 diabetes for at least 3 years and if they were treated with oral antidiabetics or insulin. For the DiabOS Study patients and non-diabetic controls were divided into groups with (T2DMFx, CoFx) and without fragility fractures (T2DM and Co). Participants needed to be able to move without assistance.

Exclusion criteria comprised any medical or psychiatric condition which would preclude the participants from adhering to the protocol, idiopathic or premenopausal osteoporosis, previous treatment with osteoporosis medication or intake of drugs known to affect bone metabolism (e.g. steroids, thiazolidinediones) within 6 months prior to enrolment or medical conditions known to affect bone health (e.g. metabolic bone disease such as primary hyperparathyroidism or Paget’s disease, metastatic bone disease, thyrotoxicosis, hypercortisolism).

Within the DiabOS study participants were followed at yearly intervals over 3 years (clinical, biochemical and densitometric assessment). During a standardized interview data on current medical treatment and presence of microvascular diabetic complications (retinopathy, neuropathy, nephropathy defined as microalbuminuria) were obtained and supplemented by medical records when appropriate. Daily calcium intake was calculated based on a semiquantitative in-house food frequency questionnaire.

For the purpose of this cross-sectional study, the baseline biochemical data of the first 202 consecutive participants without fragility fractures recruited for DiabOS were evaluated including 110 patients with T2DM (median disease duration, 13.5 years) and 92 non-diabetic controls.

### Biochemical Assessment

Fasting blood samples were drawn between 08:00 and 11:00 a.m. and fresh serum aliquots were stored at −80 °C until analysis. Samples were analysed for HbA1c (Alere, Afinion), fasting glucose and insulin with the automated Elecsys® Insulin assay on a cobas e 411 analyzer (Roche Diagnostics International, Rotkreuz, Switzerland).

Calcium, phosphate, magnesium and creatinine were analysed by standard method on an autoanalyzer (Hitachi System 704 analyzer; Roche Diagnostics International, Rotkreuz, Switzerland). Serum bone-specific alkaline phosphatase (BAP) was determined by an ELISA assay (MicroVueBAP, Quidel, San Diego/USA); the intra- and interassay variations were < 5.8% and 7.6%, respectively. The parameters CTX, PINP, 25-Hydroxyvitamin D (25OH vitamin D, Vitamin D total) and intact parathyroid hormone (iPTH) were measured in serum with Elecsys® assays on the automated analyzer cobas e 411 (Roche Diagnostics International, Rotkreuz, Switzerland). The intra- and interassay variations were 2.0 and 8.4% for CTX, 1.2 and 3.3% for PINP, 2.2 and 10.7% for 25OH vitamin D and 1.2 and 2.0% for iPTH, respectively. The expected values for the iPTH assay were 15–65 pg/mL (1.6–6.9 pmol/L) according to the manufacturer. Intact PTH was assessed by a second generation assay which although it detects inactive fragments of PTH alongside the active fragment is still the preferred reliable assay with the most clinical experience [18].

Serum sclerostin was determined in duplicate by an enzyme immunoassay; the intra- and interassay variations were ≤ 7 and ≤ 10%, respectively (Biomedica, Vienna/Austria).

Urinary albumin was measured by immunoturbidimetry [MicroAlb SeraPak (Bayer, Tarrytown, NY) on Cobas Mira Plus (Roche Diagnostics International, Rotkreuz, Switzerland)]. The mean intra- and inter-assay variations were 4.5 and 7.6%, respectively. Urine creatinine and urine phosphate were measured on an automated analyser (Beckman Coulter® AU analyzer). Renal tubular reabsorption of phosphate (Tmp/GFR in mmol/l of glomerular filtrate) was calculated using the following equation according to Payne RB: TmP/GFR = TRP x serum phosphate. TRP (fractional tubular reabsorption of phosphate) = 1 – (Serum creatinine x Urine phosphate/Urine creatinine x Serum phosphate) [32].

### Statistical Analysis

Descriptive statistics of categorical parameters were summarized as counts and percentages (e.g. gender, comorbidities, insulin treatment, neuropathy, retinopathy, nephropathy). Chi-square test or the Fisher’s exact test were used to compare the T2DM and control groups. Continuous parameters were expressed as means and standard deviations (SD) or medians and interquartile range (IQR). Mann–Whitney U test was used to compare the two groups. The means were adjusted for age, gender and BMI. Serum magnesium levels were additionally adjusted for thiazide and proton pump inhibitor use. When adjusting the means generalized linear regression models were used and the respective adjusted means were extracted.

A multivariate regression analysis was done for PINP, CTX and BAP. Furthermore, correlation analyses were calculated between iPTH and HbA1c, disease duration, serum bone turnover markers, urinary albumin/creatinine ratio, magnesium and vascular complications. Pearson correlation coefficients were calculated for correlation with continuous parameters, and Spearman´s correlation coefficients were calculated for correlations with categorical and continuous parameters.

The software Statistical Analysis System 9.4 (SAS Institute, Cary NC, USA) was used for the analysis. The level for statistical significance was set to an alpha < 0.05.

## Results

### Patient Characteristics

In this cross-sectional case–control study we included 110 patients with T2DM and 92 non-diabetic controls. Patients with T2DM were predominantly male (T2DM male 71.82%, CO male 25%, *p* < 0.001), were older (T2DM 63.7 ± 6.0 yrs, CO 60.5 ± 6.3 yrs, *p* < 0.001) and had a significantly higher BMI (T2DM 29.8 ± 4.3 kg/m^2^, CO 24.9 ± 4.5 kg/m^2^, *p* < 0.001) and more comorbidities (T2DM *n* = 106/110, 96.4%, CO *n* = 74/92, 80.4%, *p* < 0.001) as compared to non-diabetic controls.

Median diabetes duration was 13.5 years (IQR 8–20 years). Diabetes was moderately to well controlled with a mean HbA1c of 7.5 ± 1.2%. 64 patients (58.2%) were treated with insulin and in 76 patients (69.1%) microvascular complications were present. 85.4% of the T2DM patients were on Metformin treatment, 23.6% were taking SLGT-2 inhibitors. (Table 1).

**Table 1:** Baseline characteristics in T2DM and non-diabetic controls (Co)

Characteristics	T2DM *n* = 110	Co *n* = 92
Female, *n* (%)*	31 (28.2)	69 (75.0)
Age (years) ^§^*	63.7 ± 6.0	60.5 ± 6.3
Body mass index (kg/m^2^)^§*^	29.9 ± 4.3	24.9 ± 4.5
HbA1c (%)^§^*	7.5 ± 1.2	5.5 ± 0.3
Disease duration (years) ^§^	13.5 (8, 20)	n/a
Insulin treatment, *n* (%)	64 (58.2)	n/a
Metformin treatment, *n* (%)	94 (85.4)	n/a
GLP-1 treatment, *n* (%)	45 (40.9)	n/a
SGLT-2 treatment, *n* (%)	26 (23.6)	n/a
DPP-4 treatment, *n* (%)	26 (23.6)	n/a
Sulfonylurea treatment, *n* (%)	11 (10.0)	n/a
Polyneuropathy, *n* (%)	40 (36.4)	n/a
Retinopathy, *n* (%)	11 (10.0)	n/a
Nephropathy^1^, *n* (%)	25 (22.7)	n/a

^**§**^Values presented as mean ± SD or median (interquartile range, IQR)

*p-value < 0.001 for comparison between T2DM and non-diabetic controls

^1^defined as microalbuminuria

### Biochemical Characteristics

**Fig. 1:**
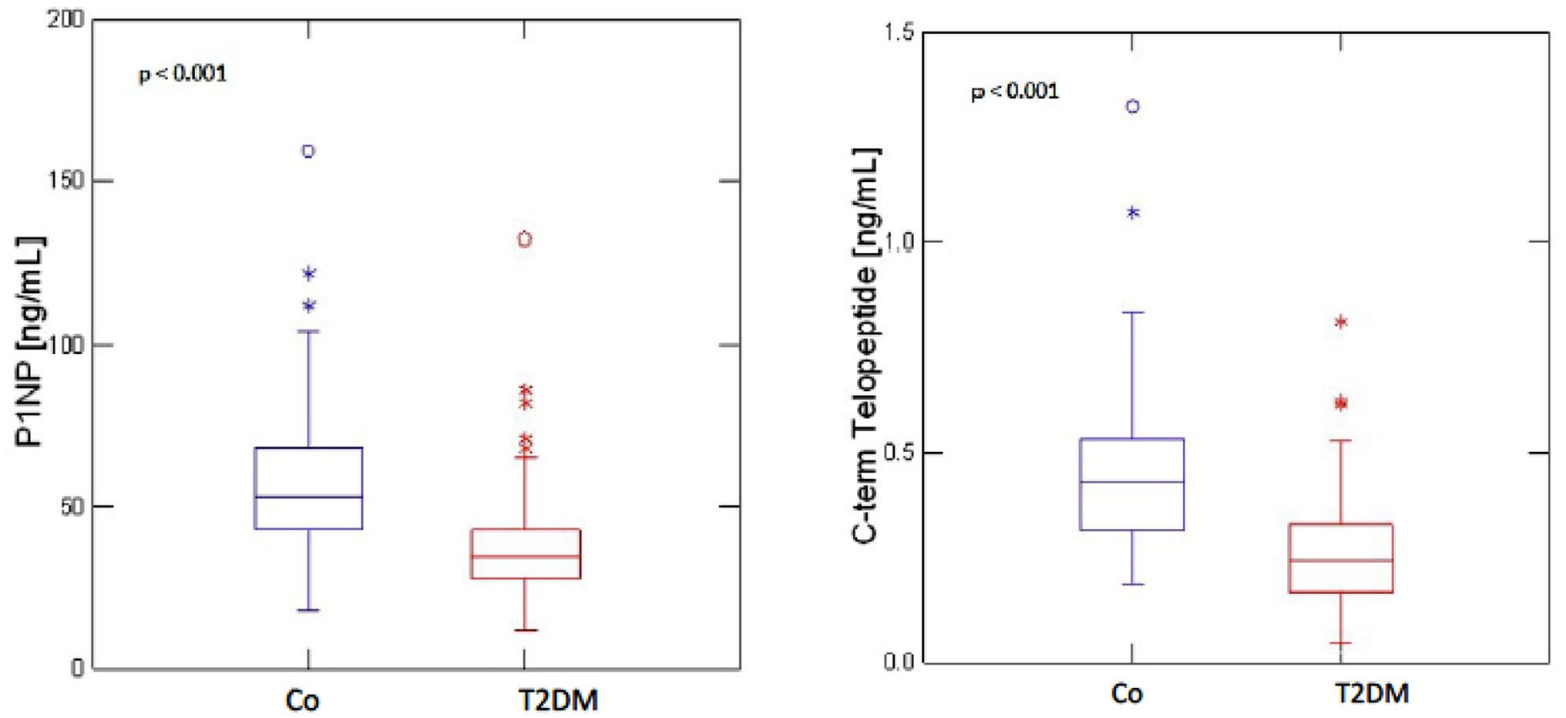
Distribution of serum PINP and CTX levels in T2DM (*n* = 110) and non-diabetic controls (Co) (*n* = 88)

**Fig. 2:**
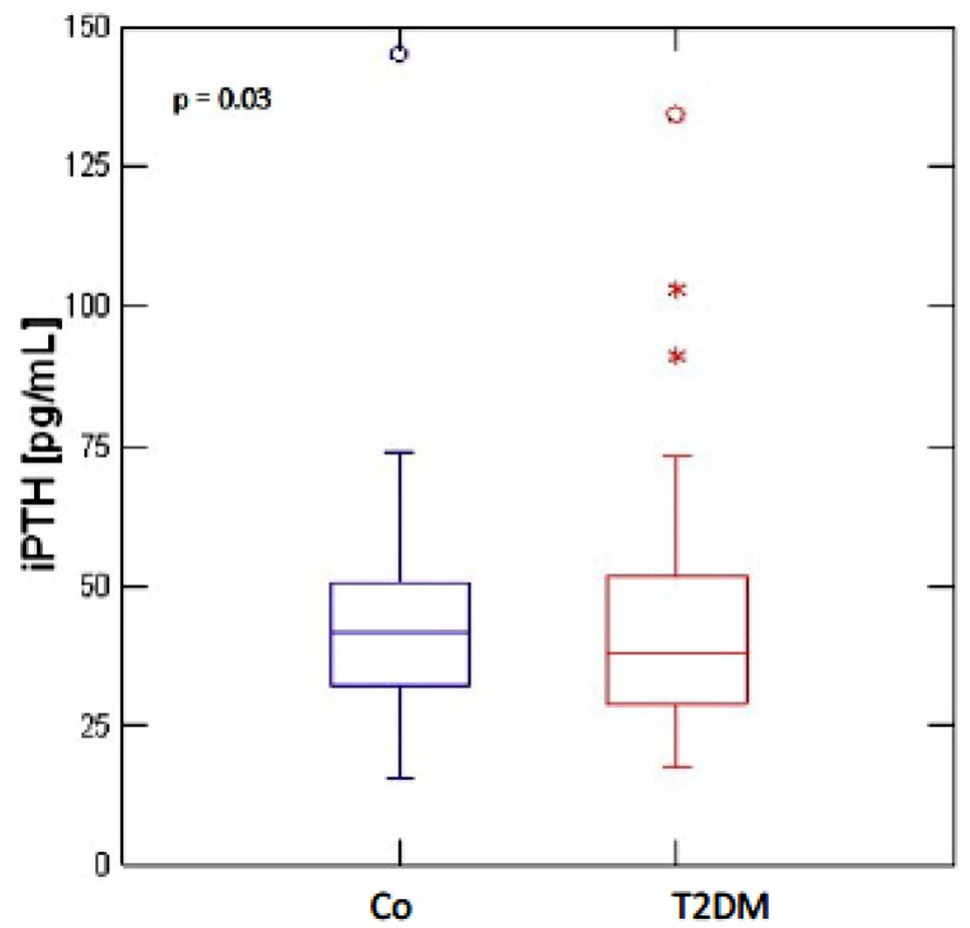
Distribution of serum intact PTH levels in T2DM (*n* = 108) and non-diabetic controls (*n* = 88)

Raw data distribution of PINP, CTX, iPTH and magnesium for T2DM and non-diabetic controls is shown in Figs. 1, 2 and3. The results after adjustment for age, gender and BMI are presented in Table 2: After adjustment patients with T2DM had significantly lower serum levels of PINP (T2DM: 39.61 ng/ml, 95%CI 35.37–43.85; CO: 57.38 ng/ml, 95%CI 52.57–62.18; *p* < 0.001) and CTX (T2DM: 0.29 ng/ml, 95%CI 0.26–0.32; CO: 0.43 ng/ml, 95%CI 0.39–0.46; *p* < 0.001) as compared to non-diabetic controls. BAP levels did not significantly differ between the groups (T2DM: 14.73 μg/l, 95%CI 13.24–16.23; CO:16.45 μg/l, 95%CI 14.75–18.14; *p* = 0.18). Serum iPTH (T2DM: 39.23 pg/ml, 95% CI 35.39–43.07; CO: 46.47 pg/ml, 95% CI 42.17–50.77; *p* = 0.03) and magnesium levels (T2DM: 0.81 nmol/L, 95%CI 0.79–0.83; CO: 0.89 nmol/L, 95%CI 0.86–0.92; *p* < 0.001) were significantly lower in T2DM. For serum magnesium levels we performed an additional adjustment for proton pump inhibitor use and thiazide use.

**Fig. 3:**
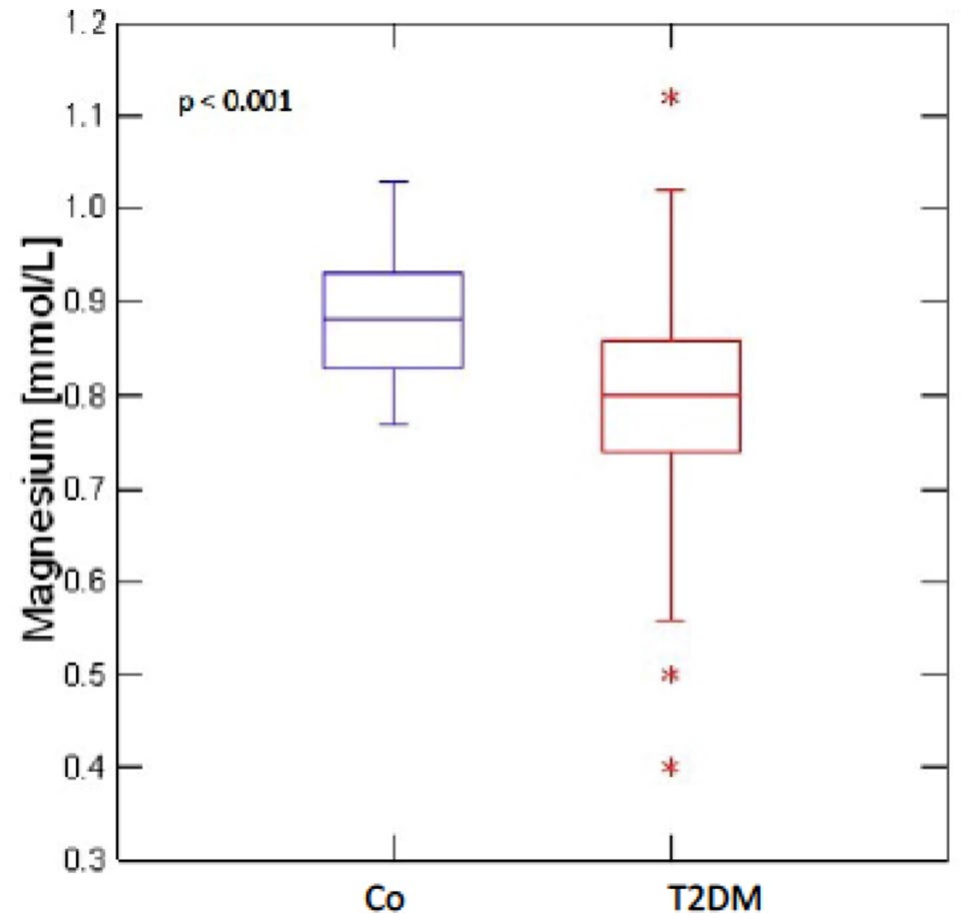
Distribution of serum magnesium levels in T2DM (*n* = 108) and non-diabetic controls (*n* = 89)

**Table 2:** Biochemical characteristics and dietary calcium and vitamin D intake in T2DM and non-diabetic controls (Co)

Characteristics	T2DM			CO			*p*
	*n*	Adjusted mean	95% CI	n	Adjusted mean	95% CI	
Fasting glucose (mmol/l)	105	7.63	7.29, 7.96	90	5.57	5.20, 5.93	** < 0.001**
PINP (ng/ml)	110	39.61	35.37, 43.85	88	57.38	52.57, 62.18	** < 0.001**
CTX (ng/ml)	110	0.29	0.26, 0.32	88	0.43	0.39, 0.46	** < 0.001**
Bone-specific alkaline phosphatase (μg/l)	110	14.73	13.24, 16.23	88	16.45	14.75, 18.14	0.18
Alkaline phosphatase (U/l)	110	68.99	64.58, 73.41	89	67.19	62.22, 72.17	0.64
Sclerostin (mmol/l)	109	39.10	36.01, 42.19	88	37.37	33.88, 40.86	0.51
Intact PTH (pg/ml)	108	39.23	35.39, 43.07	88	46.47	42.17, 50.77	0.03
25OH vitamin D (nmol/l)	110	59.00	53.60, 64.39	88	65.06	58.95, 71.18	0.19
Calcium (mmol/l)	110	2.36	2.33, 2.38	89	2.35	2.32, 2.37	0.64
Phosphorus (mmol/l)	110	1.24	1.21, 1.28	89	1.15	1.11, 1.19	**< 0.001**
Magnesium (mmol/l)*	110	0.81	0.79, 0.83	89	0.89	0.86, 0.92	**< 0.001**
Sodium (mmol/l)	110	137.74	136.96, 138.38	89	138.51	137.63, 139.98	0.24
Creatinine (μmol/l)	110	73.99	69.79, 78.19	89	74.23	69.49, 78.97	0.95
eGFR (ml/min)	110	86.44	83.25, 89.63	89	85.84	82.25, 89.44	0.83
U-albumin/creatinine ratio (mmol/mmol)	107	5.33	3.18, 7.48	86	1.89	0.56, 4.33	0.07
Tmp/GFR** (mmol/l)	108	1.12	1.09, 1.15	87	1.03	0.99, 1.06	** < 0.001**
Dietary calcium intake (mg/d)	109	877	791, 963	92	954	859, 1049	0.29
Vitamin D supplementation (IU/d)	30	1169	826–1512	34	926	566–1287	0.38

all comparisons are adjusted for age, gender and BMI

^*^additional adjustment for thiazide use and proton pump inhibitor use **renal tubular reabsorption of phosphate

Significant *p*-levels < 0.05 are highlighted in bold

Phosphate levels in the T2DM population were in the normal standard range but significantly higher (T2DM: 1.24 mmol/L, 95%CI 1.21–1.28; CO: 1.15 mmol/L, 95%CI 1.11–1.19; *p* < 0.001) than in non-diabetic controls. In line with this finding, renal tubular phosphate reabsorption (Tmp/GFR) was significantly higher in the diabetic group (*p* < 0.001).

Serum calcium, creatinine, 25OH vitamin D, nutritional calcium intake and Vitamin D supplementation did not differ between the groups after adjustment for age, gender and BMI.

Unadjusted serum sclerostin levels were higher in T2DM compared to the non-diabetic population. However, this effect did not remain significant after adjustment for age, gender and BMI (T2DM: 39.1 mmol/L, 95%CI 36.01–42.19; CO: 37.37 mmol/L, 95%CI 33.88–40.86; *p* = 0.51) (Table 2). In the adjusted model the strongest effect was due to age (*p* = 0.007) and gender (*p* < 0.0001).

### Multivariate Regression and Correlation Analyses

We investigated determinants affecting bone turnover markers in T2DM by multivariate regression analysis (Table 3). Hereby only serum iPTH remained an independent determinant of lower serum bone turnover markers in T2DM. Intact PTH was positively associated with PINP (*p* = 0.02), BAP (*p* < 0.01) and CTX (*p* < 0.001). No significant association was found between serum bone turnover markers and other parameters such as age, BMI, HbA1c, serum sclerostin and diabetes disease duration. Magnesium was not an independent determinant for bone turnover markers in the multivariate regression analysis.

**Table 3:** Multivariate regression analysis in T2DM: Parameters determining serum bone turnover markers (PINP, CTX, BAP)

Factors	Value	*n*	Outcome								
			PINP (ng/ml)			CTX (ng/ml)			BAP (µg/L)		
			Estimate	SE	*p*	Estimate	SE	*p*	Estimate	SE	*p*
Intercept	–		27.31	20.96	0.96	0.24	0.19	0.20	3.46	9.97	0.73
Age (years)	1 unit increase	101	−0.32	0.22	0.16	−0.01	0.01	0.34	0.04	0.11	0.71
BMI (kg/m^2^)	1 unit increase	101	0.62	0.31	0.05	0.01	0.01	0.23	0.04	0.15	0.81
Magnesium (mmol/l)	1 unit increase	101	6.08	12.07	0.62	0.13	0.11	0.23	−1.58	5.74	0.78
Sclerostin (mmol/l)	1 unit increase	101	0.13	0.07	0.08	0.01	0.01	0.78	−0.06	0.04	0.09
iPTH (pg/ml)	1 unit increase	101	0.16	0.69	**0.02**	0.01	0.001	**< 0.001**	0.09	0.03	**< 0.01**
HbA1c (%)	1 unit increase	101	−0.89	1.11	0.43	−0.14	0.01	0.18	1.05	0.53	0.05
Disease duration (years)	1 unit increase	101	0.07	0.19	0.72	−0.01	0.01	0.11	−0.10	0.09	0.25
Adjusted R2			0.10			0.14			0.08		

*BIC* Bayesian information criterion, *AIC* Akaike information criterion, *SE* standard error

Significant *p*-levels < 0.05 are highlighted in bold

Additional analyses showed that lower serum iPTH correlated with lower PINP (*r* = 0.31, *p* = 0.001), CTX (*r* = 0.36, *p* < 0.001) and BAP (*r* = 0.26, *p* < 0.01) in patients with T2DM but not in non-diabetic controls (Table 4A and B).

**Table 4:** A) Correlation analyses for iPTH in T2DM

Correlation of	With	*n*	Correlation coefficient	Type of correlation coefficient	*p*
iPTH	Retinopathy	108	0.06	Spearman´s	0.54
	Neuropathy	108	−0.14	Spearman´s	0.14
	Nephropathy	108	0.03	Spearman´s	0.78
	U-albumin/creatinine Ratio	107	−0.06	Pearson	0.51
	HbA1c	108	−0.01	Pearson	0.95
	Disease Duration	108	0.15	Pearson	0.12
	Magnesium	108	0.21	Pearson	**0.03**
	PINP	108	0.31	Pearson	**0.001**
	CTX	108	0.36	Pearson	**< 0.001**
	BAP	108	0.26	Pearson	**< 0.01**

Correlation of	With	*n*	Correlation coefficient	Type of correlation coefficient	*p*
iPTH	Magnesium	88	0.12	Pearson	0.26
	PINP	87	0.07	Pearson	0.53
	CTX	87	0.10	Pearson	0.38
	BAP	87	−0.05	Pearson	0.65

Significant *p*-levels < 0.05 are highlighted in bold

We also found a positive correlation between iPTH and Magnesium in diabetes only (*p* = 0.03, Table 4A). In addition, we observed significantly lower iPTH levels in T2DM with magnesium levels ≤ 0.8 mmol/l (*n* = 55; median [IQR], 33.4 pg/ml [26.4 to 47.2]) as compared to patients with magnesium levels > 0.8 mmol/l (*n* = 53; 42.0 pg/ml [32.4 to 53.9]; *p* = 0.02). In non-diabetic controls serum iPTH were comparable in subjects with lower or higher magnesium levels.

There was no significant correlation between iPTH and HbA1c in T2DM.

## Discussion

Within the present study we intended to assess potential determinants of low bone turnover in T2DM by evaluating the association between serum markers of bone resorption and formation with calciotropic hormones, serum sclerostin, glycaemic control and diabetes disease duration. Our results confirm that T2DM is associated with low bone turnover, as indicated by significantly lower levels of CTX and PINP compared to non-diabetic controls. We also found significantly lower serum iPTH levels in the diabetic group. In multivariate regression analyses low serum iPTH was shown to be an independent determinant of both bone resorption and bone formation parameters. No associations were found between serum bone turnover markers and glycaemic control, sclerostin levels or disease duration. Our results suggest that serum iPTH may be a regulator of low bone turnover in T2DM. Furthermore, patients with T2DM had significantly lower serum magnesium levels than controls and iPTH was positively correlated with serum magnesium in T2DM. We found significantly lower iPTH levels in diabetic patients with serum magnesium between 0.5 and 0.8 mmol/as compared to patients with normomagnesemia. We speculate that in the subset of T2DM patients with mild hypomagnesemia the observed relative hypoparathyroidism might be regulated by magnesium.

PTH promotes both bone formation and resorption via osteocyte signalling [33] by mechanisms which are not fully understood yet. Osteocytes are known to be crucial mediators of PTH action and play a central role in skeletal remodelling [33]. PTH reduces osteoblast apoptosis [34], inhibits adipocyte differentiation of early stem cells in the osteoblast lineage [35] and increases the number of skeletal progenitors [36].

Reyes-Garcia et al. found that decreased PTH levels in T2DM were associated with reduced serum levels of CTX and TRACP5b [22]. Dobnig et al. showed that decreased PTH levels and higher levels of glycaemia both independently contribute to lower bone turnover in elderly nursing home patients with T2DM [4]. Our patients with T2DM had significantly lower iPTH levels which were associated with lower levels of both PINP and CTX. Serum calcium and creatinine levels, 25 OH vitamin D and vitamin supplementation as well as calcium intake did not differ between the groups. We found significantly higher phosphate levels in T2DM compared to controls which may possibly be related to a relative hypoparathyroidism with increased renal phosphate reabsorption in T2DM. 23.6% of diabetic patients were taking SGLT-2 inhibitors which have been reported to be associated with higher renal phosphate reabsorption but also, unlike in our study population, higher magnesium and iPTH levels and increased bone resorption [37]. Although the classical constellation of hypoparathyroidism with hypocalcemia was not present in patients with T2DM, iPTH levels were significantly lower and this relative hypoparathyroidism was found to be an independent determinant of low bone turnover in T2DM.

Amongst other mechanisms iPTH is supposed to downregulate the SOST gene encoding for sclerostin which is an osteocyte-specific secreted WNT-inhibitor of bone formation [28, 38, 39]. Sclerostin levels increase with age and PTH was shown to be an independent determinant of sclerostin in patients without diabetes [29, 40, 41]. In type 1 diabetes (T1DM) and T2DM sclerostin levels were found to be higher compared to non-diabetic controls [10, 11, 29] which might be due to an impaired suppression of sclerostin production by PTH in patients with diabetes [29]. We hypothesized that the relative hypoparathyroidism in patients with T2DM might result in higher sclerostin levels, but in our cohort sclerostin levels were not significantly altered in T2DM. We could neither show a significant correlation between sclerostin and PTH levels nor between sclerostin and HbA1c. Although PTH is supposed to control sclerostin effects [42], it also uses other mechanisms to exert its influence on bone [33]. Our results suggest that specifically in T2DM other effects than those related to sclerostin might be responsible for low bone turnover.

The relative hypoparathyroidism described in T2DM has been linked to hyperglycaemia [4, 22, 30, 31], yet findings are inconsistent. Yamamoto et al. found low PTH levels in T2DM but did not identify any significant correlation between PTH and HbA1c [21].

Hyperglycaemia itself is known to impair osteoclast and osteoblast function in vitro [26]. Elevated plasma glucose is associated with decreased serum levels of CTX, PINP and OC [27]. In patients with diabetes negative correlations between levels of HbA1c, OC and CTX were found [16, 28]. Okazaki et al. [43] and Kanazawa et al. [5] demonstrated that OC levels increased after improvement of glycaemic control.

We did not find any correlation between HbA1c, disease duration and bone turnover markers. One possible explanation might be that our T2DM population was in average medium and well-controlled with a mean HbA1c of 7.5% (± 1.2), whereas in the aforementioned studies [5, 21, 43] patients with diabetes had worse glycaemic control with an average HbA1c of at least 8.9% (± 2.5) [5].

As other possible mechanisms for an impaired PTH secretion in patients with T2DM a calcium-sensing defect and chronic hypomagnesemia [23] were previously described. We found significantly lower serum magnesium levels in our diabetic patients in comparison to non-diabetic controls which remained significant after correction for PPI and thiazide use. A substantial proportion (85.4%) of our diabetic patients were on metformin treatment which has been linked to hypomagnesemia in observational cohort studies [44]. However, a recent in vivo study showed that metformin treatment hat no effect on magnesium homeostasis in diabetic mice which led to the conclusion that hypomagnesemia is a consequence of T2DM which is not modulated by metformin intake [45].

Interestingly, we saw a significant, albeit weak positive correlation between PTH and magnesium levels in T2DM which was not present in healthy controls.

PTH secretion by the parathyroid gland is physiologically controlled by serum calcium levels, but magnesium can exert similar effects. An increase in extracellular magnesium inhibits PTH secretion by activation of the calcium-sensing receptor [46]. Whilst low levels of magnesium are known to stimulate PTH secretion by activation of the calcium-sensing receptor, only severe hypomagnesemia with serum magnesium < 0.5 mmol/l and an intracellular magnesium deficit is supposed to induce a paradoxical block of PTH secretion [46, 47]. Accordingly, we did not find any positive correlation between magnesium and PTH in our non-diabetic controls with normal magnesium and iPTH levels. An in vitro study demonstrated that rat parathyroid glands are sensitive to an inhibitory effect of magnesium only in the presence of a moderate to low calcium concentration [48]. We speculate that the lack of correlation between magnesium and iPTH in our controls may be related to a calcium level within the normal range.

In contrast, in T2DM, albeit calcium levels were not significantly different from the control group, iPTH levels were lower in patients with moderately low magnesium levels < 0.8 mmol/l: We therefore speculate that unlike in the control group lower magnesium levels in T2DM might be associated with an impaired PTH secretion.

The prevalence of hypomagnesemia is increased in T2DM especially in patients with poor glycaemic control, with a longer disease duration and with chronic vascular complications [24, 25, 49]. It has been shown that the net tubular reabsorption of magnesium is decreased in diabetic patients in the presence of hyperglycaemia, leading to hypermagnesiuria and hypomagnesemia [50]. Furthermore, PTH in turn regulates magnesium homeostasis by modulating renal magnesium reabsorption through the distal convoluted tubule [23].

We speculate that the observed relative hypoparathyroidism in T2DM is mediated by lower magnesium levels in the subset of patients with mild hypomagnesemia. Hypoparathyroidism might also induce hypermagnesiuria which in turn could worsen the hypomagnesemic state in T2DM.

This study has particular limitations. First, the analyses are cross-sectional and can show only associations. Furthermore, our T2DM population was small and heterogenous, with more men in the diabetic group and more women in the control group. Although these differences were corrected for in the baseline characteristics, the small sample size and heterogeneity reduce the statistical power of this study. Furthermore, the mentioned results were significant but altogether weak, so that under consideration of the reduced statistical power they can only be used to generate hypotheses.

In conclusion, we confirm that T2DM is a state of low bone turnover. Low serum iPTH appears to be a regulator of low bone turnover in T2DM, whereas glycaemic control and disease duration seem not to be associated. Based on the relationship between serum levels of iPTH and magnesium, we hypothesize that in the cohort of T2DM with mild magnesium depletion low bone turnover may be linked to a hypomagnesaemia-related functional hypoparathyroidism. However, the mechanism underlying low iPTH in T2DM in general remains to be elucidated. Future investigations are required to (a) further explore the influence of relative hypoparathyroidism on bone turnover in T2DM and (b) to assess whether measurements of serum iPTH and magnesium are of clinical value in patients with type 2 diabetes.
